# Hypolipidemic Effects and Safety of *Lactobacillus Reuteri* 263 in a Hamster Model of Hyperlipidemia

**DOI:** 10.3390/nu7053767

**Published:** 2015-05-15

**Authors:** Wen-Ching Huang, Yi-Ming Chen, Nai-Wen Kan, Chun-Sheng Ho, Li Wei, Ching-Hung Chan, Hui-Yu Huang, Chi-Chang Huang

**Affiliations:** 1 Graduate Institute of Athletics and Coaching Science, National Taiwan Sport University, Taoyuan 33301, Taiwan; E-Mails: 1010503@ntsu.edu.tw (W.-C.H.); kevinkan@tmu.edu.tw (N.-W.K.); 2 Graduate Institute of Sports Science, National Taiwan Sport University, Taoyuan 33301, Taiwan; E-Mail: 1021302@ntsu.edu.tw; 3 Center for Liberal Arts, Taipei Medical University, Taipei 11031, Taiwan; 4 College of Exercise and Health Sciences, National Taiwan Sport University, Taoyuan 33301, Taiwan; E-Mail: 1031213@ntsu.edu.tw; 5 Division of Physical Medicine and Rehabilitation, Lo-Hsu foundation, Inc., Lotung Poh-Ai Hospital, Yilan 26546, Taiwan; 6 Department of Neurosurgery, Taipei Medical University-WanFang Hospital, Taipei 11696, Taiwan; E-Mail: nsweili@gmail.com; 7 Department of Food Science, Nutrition, and Nutraceutical Biotechnology, Shih Chien University, Taipei 10462, Taiwan; E-Mail: llfonly_520@hotmail.com

**Keywords:** hypolipidemic, cholesterol, triglyceride, high-cholesterol diet, lipid-lowering

## Abstract

We aimed to verify the beneficial effects of probiotic strain *Lactobacillus reuteri* 263 (Lr263) on hypolipidemic action in hamsters with hyperlipidemia induced by a 0.2% cholesterol and 10% lard diet (*i.e.*, high-cholesterol diet (HCD)). Male Golden Syrian hamsters were randomly divided into two groups: normal (*n* = 8), standard diet (control), and experimental (*n* = 32), a HCD. After a two-week induction followed by a six-week supplementation with Lr263, the 32 hyperlipidemic hamsters were divided into four groups (*n* = 8 per group) to receive vehicle or Lr263 by oral gavage at 2.1, 4.2, or 10.5 × 10^9^ cells/kg/day for 6 weeks, designated the HCD, 1X, 2X and 5X groups, respectively. The efficacy and safety of Lr263 supplementation were evaluated by lipid profiles of serum, liver and feces and by clinical biochemistry and histopathology. HCD significantly increased serum levels of total cholesterol (TC), triacylglycerol (TG) cholesterol, high-density lipoprotein cholesterol (HDL-C), and low-density lipoprotein cholesterol (LDL-C), LDL-C/HDL-C ratio, hepatic and fetal TC and TG levels, and degree of fatty liver as compared with controls. Lr263 supplementation dose dependently increased serum HDL-C level and decreased serum TC, TG, LDL-C levels, LDL-C/HDL-C ratio, hepatic TC and TG levels, and fecal TG level. In addition, Lr263 supplementation had few subchronic toxic effects. Lr263 could be a potential agent with a hypolipidemic pharmacological effect.

## 1. Introduction

Hyperlipidemia is a widely known key risk factor for cardiovascular diseases. High blood cholesterol and triacylglycerol levels are commonly considered important modulators and biomarkers of hyperlipidemic processes [1]. Therefore, the management of these two parameters is necessary for cardiovascular health. Probiotic bacteria are defined by the World Health Organization (WHO) as “live microorganisms which when administered in adequate amounts confer a health benefit on the host” and are being examined for their efficacy in lowering total cholesterol (TC) and low-density lipoprotein cholesterol (LDL-C) in humans [2]. Intestinal lactic acid bacterial (LAB) species with alleged health beneficial properties have been introduced as probiotics. LAB species are important members of the normal intestinal microflora and showed beneficial effects in study of the molecular biology and genomics of Lactobacillus in immune function, anti-cancer, and antibiotic-associated diarrhea, travelers’ diarrhea, pediatric diarrhea, inflammatory bowel disease and irritable bowel syndrome [3].

*Lactobacillus* spp. occurs in the gastrointestinal ecosystem of humans, poultry, swine, and other animals. They are excellent probiotic microorganisms because of their activities in ameliorating enteric diseases, maintaining health, and inhibiting melanin synthesis [4,5]. *Lactobacillus* *reuteri* produces a broad-spectrum antimicrobial substance during fermentation of glycerol, which revealed that glycerol fermentation was associated with the production of beta-hydroxypropionic acid and trimethylene glycol [6]. *L.* *reuteri* is used as a probiotic for chronic constipation [7], inhibits *Helicobacter pylori* load in humans [8], and removes cholesterol [2].

Previous studies used the hamster model to evaluate the hypolipidemic effect because it has many similarities with human fat-induced atherosclerotic disease. As for humans, hamsters are endowed with cholesterol ester transfer protein and all of the enzymatic pathways in lipoproteins and bile metabolism; atherosclerotic plaques develop in response to a fat diet in lesion-prone areas similar to humans [9,10,11].

*L. reuteri* 263 is a patented strain for improving the syndrome of diabetes (US 20110300117 A1) and renal fibrosis in diabetes (US 20120183504 A1), which is different from other strains such as *L. reuteri* L3 for preventing obesity in obese mice [12] or the *L. reuteri* LR6-fermented product for controlling hyperlipidemia in rats [13]. In addition, species of the same bacterial strains or even strains of the same species may feature different biological functions [12]. Qiao *et al.* demonstrated that *L. reuteri* L3 but not *L. reuteri* L10 had anti-inflammation and anti-obesity properties for obese mice [12]. Because of the complexity of host-bacterial cross-talk and the importance of investigating specific bacterial strains, we conducted experiments to evaluate the therapeutic effectiveness of *L. reuteri* 263 supplementation on the regulation of hyperlipidemia in a dyslipidemic hamster model. We also examined the biochemical parameters and liver tissues by histopathology.

## 2. Experimental Section

### 2.1. Materials, Animals, and Experiment Design

*L. reuteri* 263 (Lr263) was obtained from GenMont Biotech Inc. (Tainan, Taiwan). The dose of Lr263 for humans is 900 mg per day (lyophilized powder), equivalent to a daily recommended dose at 2.1 × 10^9^ cells/serving/day. The hamster dose (111 mg/kg) was converted from a human equivalent dose (HED) based on body surface area by the following formula from the US Food and Drug Administration: assuming a human weight of 60 kg, the HED for 900 (mg)/60 (kg) = 15 × 7.4 = 111 mg/kg; the conversion coefficient 7.4 was used to account for differences in body surface area between hamster and human as we recently described [14].

Specific pathogen-free male Golden Syrian hamsters (12 weeks old) were purchased from the National Laboratory Animal Center, Taipei City, Taiwan. Animals were housed in the animal facility at National Taiwan Sport University at room temperature (22 ± 1 °C) and 50% to 60% relative humidity, with a 12 h light-dark cycle (light on 7:00 AM). Distilled water and standard laboratory chow diet (No. 5001; PMI Nutrition International, Brentwood, MO, USA) were provided *ad libitum*. Before the experiments, the hamsters were acclimatized for 1 week to the environment and diet. The Institutional Animal Care and Use Committee (IACUC) of National Taiwan Sport University (NTSU) approved all animal experimental protocols, and the study conformed to the guidelines of the protocol IACUC-10307 approved by the IACUC ethics committee.

**Figure 1:**
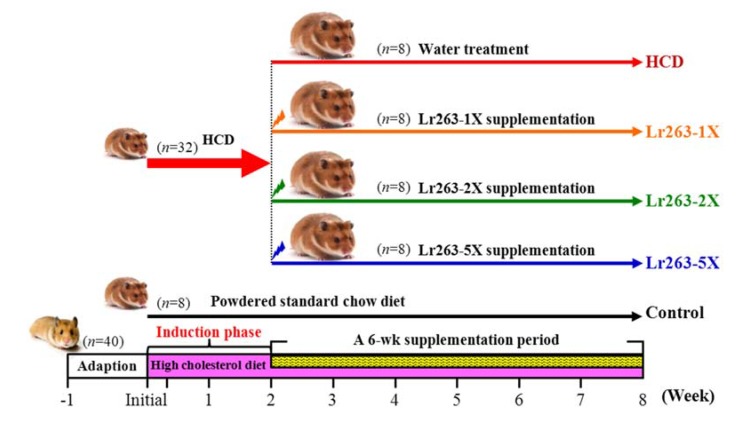
Experimental design. Control: Healthy hamsters were fed a standard laboratory diet and orally received the same volume of solution equivalent to body weight (BW). HCD: Hyperlipidemic hamsters were fed a high-cholesterol diet (HCD) and orally received the same volume of solution equivalent to BW. Lr263-1X: Hyperlipidemic hamsters were fed an HCD and orally received 111 mg/kg/day Lr263. Lr263-2X: Hyperlipidemic hamsters were fed an HCD and orally received 222 mg/kg/day Lr263. Lr263-5X: Hyperlipidemic hamsters were fed an HCD and orally received 555 mg/kg/day Lr263.

The experimental design is in Figure 1. After one-week acclimatization, 40 hamsters were divided randomly into two groups: normal (*n* = 8), fed a standard chow diet (control), and experimental (*n* *=* 32), fed a high-cholesterol diet (HCD) containing 0.2% cholesterol and 10% lard diet. The 32 resulting hyperlipidemic hamsters were divided into four groups (*n* = 8/each group): (1) HCD with vehicle (water) treatment (HCD); (2) HCD with 111 mg/kg Lr263 (Lr263-1X); (3) HCD with 222 mg/kg Lr263 (Lr263-2X); (4) HCD with 555 mg/kg Lr263 (Lr263-5X). The vehicle group received the same volume of solution equivalent to body weight (BW). The food intake and water consumption were monitored daily, and BW was recorded weekly.

### 2.2. HCD Composition

Hamsters were fed a standard chow diet or an HCD adapted from previous study [15] with some modification. The standard chow (No. 5001) contained 3.35 kcal/g with 28.5% as protein, 13.5% as fat and 58.0% as carbohydrates. The HCD contained 0.2% (wt/wt) cholesterol (Sigma-Aldrich, St. Louis, MO, USA), 10% (wt/wt) lard (Sigma-Aldrich) and 89.8% (wt/wt) standard chow, for 3.92 kcal/g with 21.96% as protein, 33.37% as fat and 44.67% as carbohydrates.

### 2.3. Liver and Fecal Lipid Analysis

Liver and fecal matter were collected after hamsters were killed. Hepatic and fecal TG and TC levels were measured in triplicate by using commercial enzymatic kits for TG (No. 10010303) and for TC (No. 10007640) from Cayman Chemical (Ann Arbor, MI, USA).

### 2.4. Clinical Biochemical Profiles

At the end of the experimental period, all hamsters were killed with 95% CO_2_ asphyxiation, and blood was immediately collected. Serum was collected by centrifugation and the clinical biochemical variables including aspartate aminotransferase (AST), alanine aminotransferase (ALT), lactate dehydrogenase (LDH), total protein (TP), blood urea nitrogen (BUN), creatinine, and glucose were measured by use of an autoanalyzer (Hitachi 7060, Tokyo, Japan).

### 2.5. Histological Staining of Tissues

Liver tissues were carefully removed, minced and fixed in 10% formalin. All samples were embedded in paraffin and cut into 4-μm thick slices for morphological and pathological evaluations. Tissue sections were stained with hematoxylin and eosin (H&E) and examined under a light microscope equipped with a CCD camera (BX-51, Olympus, Tokyo, Japan) by a veterinary pathologist.

### 2.6. Statistical Analysis

All data are expressed as mean ± SD. Statistical differences were analyzed by one-way ANOVA and the Cochran-Armitage test for trend analysis of dose-effect of Lr263 supplementation with use of SAS 9.0 (SAS Inst., Cary, NC, USA). *p* < 0.05 was considered statistically significant.

## 3. Results and Discussion

### 3.1. Hamster BW and Daily Intake

**Figure 2:**
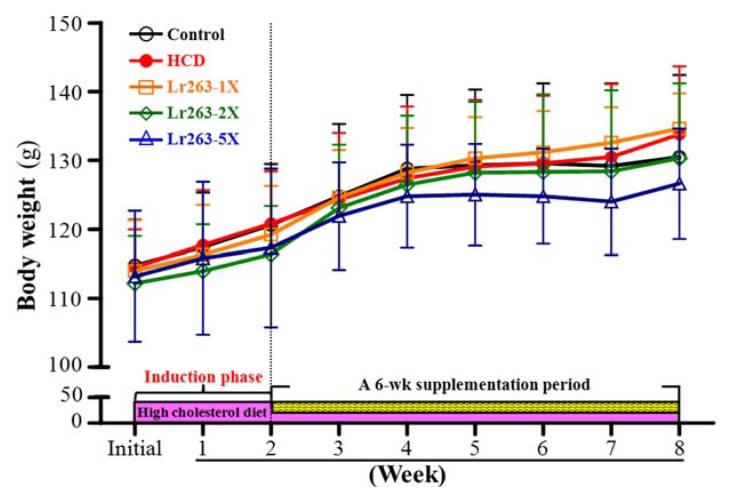
Change in BW during the experiment. The first week was an adaption phase. At two weeks, animals were separated into the control group and fed a standard laboratory diet (eight hamsters) or experimental animals and fed an HCD of the standard laboratory diet supplemented with 0.2% cholesterol and 10% lard (32 hamsters). Serum triglycerides (TG) and total cholesterol (TC) levels were higher for HCD than control hamsters. The 32 hamsters were randomly assigned to three groups (eight hamsters/group) for Lr263 supplementation. Data are mean ± SD, *n* = 8 per group.

The growth curves for hamsters are in Figure 2. In the adaption and induction phase, BW was stable and steadily increased in each group. Hyperlipidemic and control hamsters did not differ in BW at the initial and induction phases and the end of the experiment. Therefore, the HCD did not affect BW. With Lr263 supplementation, the BW curve was still stable and steadily increased, with no significant differences among groups.

The daily intake of hamsters is in Table 1. The groups did not differ in intake in the adaption and induction phases and Lr263 supplementation period. Thus, BW and daily intake of hamsters increased normally and did not differ among the groups.

**Table 1:** Body weight (BW) and daily food intake for the experimental groups.

Characteristics	Control	HCD	Lr263-1X	Lr263-2X	Lr263-5X	Trend Analysis
Initial BW (g)	115 ± 7	114 ± 6	114 ± 7	112 ± 7	113 ± 9	0.7344
Final BW (g)	130 ± 12	134 ± 10	135 ± 5	130 ± 11	127 ± 8	0.0195
*Adaption phase* Deit intake (g/hamster/day)	8.02 ± 0.75	7.95 ± 1.04	8.00 ± 0.73	7.99 ± 0.69	8.05 ± 0.52	0.8111
*Induction phase* Deit intake (g/hamster/day)	9.08 ± 0.19	9.04 ± 0.45	9.09 ± 0.35	9.09 ± 0.32	9.01 ± 0.33	0.8223
*Lr263 Supplementation* Deit intake (g/hamster/day)	8.06 ± 0.68	8.23 ± 0.88	8.06 ± 0.94	8.13 ± 0.75	8.22 ± 0.68	0.8348

Data are mean ± SD, *n* = 8 hamsters in each group. HCD: Hyperlipidemic hamsters fed a high-cholesterol diet (HCD) and orally received the same volume of solution equivalent to BW; Lr263-1X: Hyperlipidemic hamsters fed an HCD and orally received 111 mg/kg/day *Lactobacillus reuteri* 263 (Lr263). Lr263-2X: Hyperlipidemic hamsters fed an HCD and orally received 222 mg/kg/day Lr263. Lr263-5X: Hyperlipidemic hamsters fed an HCD and orally received 555 mg/kg/day Lr263.

### 3.2. Effect of Two-Week HCD Induction on Serum TC and TG Levels

**Figure 3:**
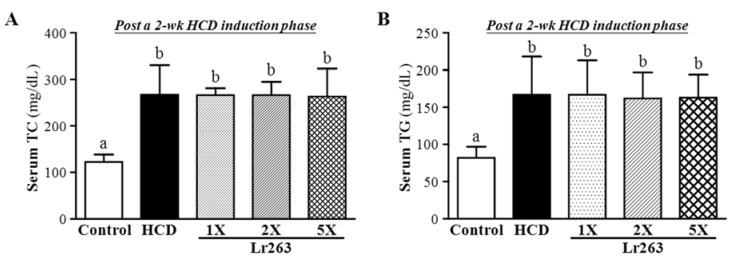
Effect of two-week HCD on serum levels of TC (**A**) and TG (**B**) in hamsters. Data are the mean ± SD, *n* = 8 hamsters in each group. Columns with different letters (a, b) significantly differ at *p* < 0.05 by a one-way ANOVA.

Serum TG and TC levels in the two weeks after induction significantly differed among groups (F(4,35) = 7.68, *p* < 0.05, η2 = 0.468; F(4,35) = 18.61, *p* < 0.05, η2 = 0.68, respectively) (Figure 3). TG and TC levels were higher with the HCD alone (before Lr263 treatment), by 1.98- to 2.18-fold (*p* < 0.0005), than controls. Supplementation with an HCD for two weeks could significantly increase the serum TC and TG levels, for an animal model of hyperlipidemia.

The mouse, rat, golden hamster, guinea pig, rabbit, pigeon and quail are often used for a hyperlipidemia disease model. Previous studies have shown that the hamster model is similar to humans in lipid metabolism (e.g., in synthesis and secretion of cholesterol). The model possesses superior efficacy in preclinical evaluation, whereas in models of rats, mice, pigeons and quails, lipoprotein metabolism differs from that in humans [16,17]. Hamsters may be a better animal model for hypercholesterolemia because the content of hyperlipidemia is easily maintained with high-fat, HCD induction [10,18]. Therefore, the hamster model has been often used to study hyperlipidemia [19,20,21].

### 3.3. Effect of Six-Week Supplementation with Lr263 on Serum Lipid Levels and LDL-C/HDL-C Ratio in Hyperlipidemic Hamsters

**Figure 4:**
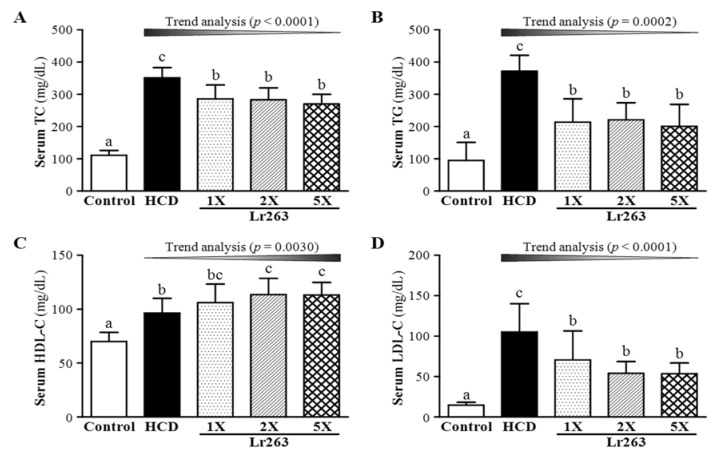

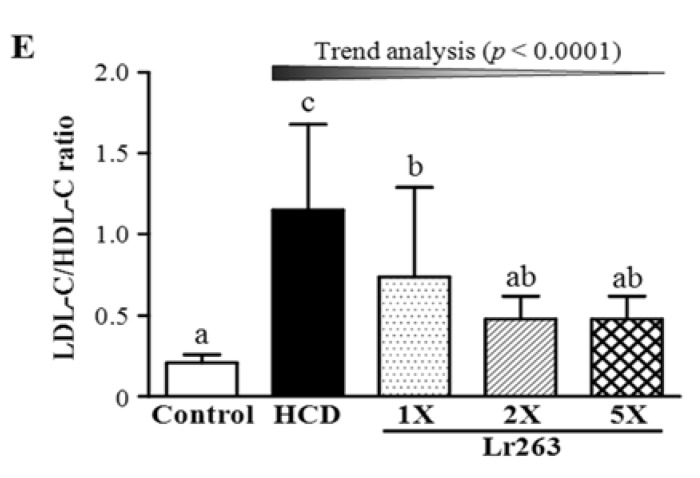
Effect of six-week supplementation with Lr263 on serum TC (**A**); TG (**B**); HDL-C (**C**); LDL-C (**D**) levels; and LDL-C/HDL-C ratio (**E**) in hyperlipidemic hamsters. Data are mean ± SD, *n*-8 hamsters in each group. Columns with different letters (a, b, c) differ significantly at *p* < 0.05 by a one-way ANOVA.

At six weeks after Lr263 supplementation, TC levels significantly differed among groups (F(4,35) = 58.75, *p* < 0.05, η2 = 0.87) (Figure 4A). TC levels were lower with Lr263-1X, Lr263-2X and Lr263-5X (18.6% (*p* = 0.0003), 19.5% (*p* = 0.0002) and 23.1% (*p* < 0.0001), respectively) than with HCD alone. Serum TG levels differed among groups (F(4,35) = 21.60, *p* < 0.05, η2 = 0.712), with levels higher for the HCD group, by 3.15-fold (*p* < 0.0001), than controls (Figure 4B). However, TG levels were lower with Lr263-1X, Lr263-2X and Lr263-5X [42.4% (*p* < 0.0001), 40.6% (*p* < 0.0001) and 45.9% (*p* < 0.0001), respectively] than with HCD alone. On trend analysis, TC and TG content was increased dose-dependently with Lr263 treatments under HCD-induced hyperlipidemia. Therefore, Lr263 can reduce serum TC and TG levels in the hyperlipidemic hamster *L.* *reuteri* during fermentation to produce glycerol. A previous study found a relationship between glycerol release and periovarian fat weight; the authors demonstrated that glycerol release decreased with increased blood free fatty acid, TG, and insulin [22]. In relevant *Lactobacillus* spp. supplementation studies, oral administration of probiotics significantly lowered cholesterol levels by as much as 22% to 33% in humans [23] or prevented elevated cholesterol levels in mice fed a fat-enriched diet [24].

At the end of the experiment, the high-density lipoprotein cholesterol (HDL-C) levels were significantly different among groups (F(4,35) = 14.39, *p* < 0.05, η2 = 0.622) (Figure 4C,D). HDL-C levels were higher with HCD alone and with 1X, 2X, and 5X Lr263, by 1.38- (*p* = 0.0004), 1.51- (*p* < 0.0001), 1.62- (*p* < 0.0001) and 1.62-fold (*p* < 0.0001) than controls. Furthermore, HDL-C levels were higher with 2X and 5X Lr263 than HCD alone (*p* = 0.0153 and *p* = 0.0175, respectively). In addition, serum LDL-C levels differed among groups (F(4,35) = 14.92, *p* < 0.05, η2 = 0.63). LDL-C levels were lower with HCD combined with Lr263-1X, Lr263-2X and Lr263-5X treatments (33% (*p* < 0.0001), 48.7% (*p* < 0.0001), and 49.1% (*p* = 0.0023), respectively) than HCD alone. On trend analysis, HDL-C and LDL-C was dose-dependently altered with Lr263 supplementation (*p* = 0.003 and *p* < 0.0001, respectively). In a previous study, an HCD could enhance serum HDL-C and LDL-C levels in the same hamster model [25]. Thus, LR263 could have modulatory effects on LDL-C levels, for a potential pharmacological effect on hyperlipidemia.

In a previous study, several mechanisms for cholesterol removal by *Lactobacillus* spp. have been proposed; one is deconjugation of bile salts by bile-salt hydrolase (BSH) assimilation of cholesterol into bacterial cell membranes to reduce cholesterol level [26]. The ability of probiotic strains to hydrolyze bile salts has often been included among the criteria for probiotic strain selection, and a number of BSHs have been identified and characterized [27]. Oral administration of *Lactobacillus* spp. was found to significantly reduce cholesterol levels, with no significant improvement in LDL-C/HDL-C ratio [28]. Therefore, different lactobacillus strains may have different cholesterol-lowering abilities.

The ratio of LDL-C/HDL-C is a criterion for evaluating the efficiency of cholesterol-lowering capacity. If the ratio is low, atherosclerotic risk factors are decreased [29]. The ratio of LDL-C/HDL-C calculated from individual hamsters differed among groups (F(4,35) = 8.14, *p* < 0.05, η2 = 0.482) and was higher for HCD alone, by 5.47-fold (*p* < 0.0001), than controls (Figure 4E). The ratio of LDL-C/HDL-C was lower with LR263-1X, LR263-2X and LR263-5X [36.1% (*p* = 0.0234), 58.0% (*p* = 0.0005) and 58.1% (*p* = 0.0005), respectively] than with HCD alone. On trend analysis, the ratio of LDL-C/HDL-C was dose-dependently decreased with Lr263 supplementation (*p* < 0.0001). In a previous study, a probiotic mix was found to modulate apolipoprotein synthesis. The mechanism was via a coordinated enterohepatic action that might be mediated by PPAR gamma/FXR upregulation [30]. In the current study, Lr263 could ameliorate cholesterol levels and improve the LDL-C/HDL-C ratio under HCD diet-induced hyperlipidemia in hamsters.

### 3.4. Effect of Six-Week Supplementation with Lr263 on Hepatic TC and TG Levels in Hyperlipidemic Hamsters

**Figure 5:**
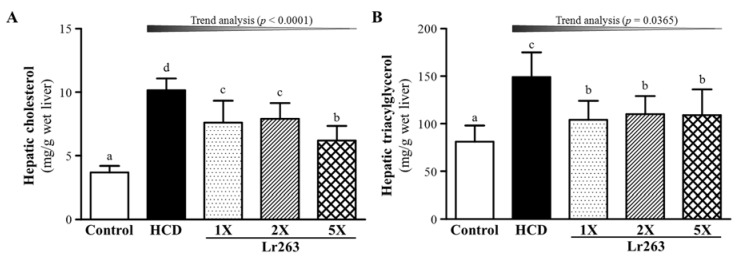
Effect of six-week supplementation with Lr263 on hepatic TC (**A**) and TG (**B**) levels in hyperlipidemic hamsters. Data are the mean ± SD, *n* = 8 hamsters in each group. Columns with different letters (a, b, c, d) significantly differ at *p* < 0.05 by a one-way ANOVA.

Liver TC content significantly differed among groups (F(4,35) = 32.82, *p* < 0.05, η2 = 0.789) and was higher with HCD alone, by 2.67-fold (*p* < 0.0001), than controls (Figure 5A). Furthermore, liver TC level was lower with Lr263-1X, Lr263-2X and Lr263-5X (25.1% (*p* = 0.0001), 22.2% (*p* = 0.0005) and 39.0% (*p* < 0.0001), respectively) than HCD alone.

Liver TG content was significantly different among groups (F(4,35) = 9.59, *p* < 0.05, η2 = 0.523) and was higher with HCD, by 1.83-fold (*p* < 0.0001), than controls (Figure 5B). Liver TG content was lower with Lr263-1X, Lr263-2X and Lr263-5X (30.3% (*p* = 0.0003), 26.1% (*p* = 0.0014) and 26.9% (*p* < 0.001), respectively) than HCD alone. On trend analysis, liver TC and TG content dose-dependently decreased with Lr263 supplementation (*p* < 0.0001 and *p* = 0.0365, respectively). Lr263 supplementation could significantly mitigate the increased liver TC and TG content induced by the HCD hyperlipidemia model.

### 3.5. Effect of Six-Week Supplementation with Lr263 on Fecal TC and TG Levels in Hyperlipidemic Hamsters

**Figure 6:**
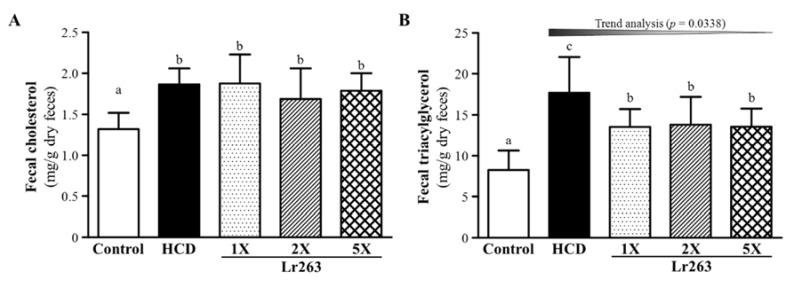
Effect of six-week supplementation with Lr263 on fecal TC (**A**) and TG (**B**) levels in hyperlipidemic hamsters. Data are mean ± SD, *n* = 8 hamsters in each group. Columns with different letters (a, b, c) significantly differ at *p* < 0.05 by a one-way ANOVA.

Fecal TC content differed among groups (F(4,35) = 5.70, *p* < 0.05, η2 = 0.395) and was higher with HCD alone, by 1.42-fold (*p* = 0.0003), than controls (Figure 6A). Fecal TC content did not differ by Lr263 supplementation. Therefore, the HCD could significantly increase the fecal TC level in all cholesterol-treated groups.

The fecal TG levels significantly differed among groups (F(4,35) = 9.95, *p* < 0.05, η2 = 0.532) and was higher with HCD alone, by 2.14-fold (*p* < 0.0001), than controls (Figure 6B). Furthermore, fecal TG content was lower with Lr263-1X, Lr263-2X and Lr263-5X, [23.7% (*p* = 0.0088), 22.1% (*p* = 0.0141) and 23.5% (*p* = 0.0092), respectively] than HCD alone. On trend analysis, Lr263 supplementation showed dose-dependently decreased fecal TG content (*p* = 0.0338). Therefore, our HCD could increase both serum and fecal TG levels. Lr263 treatment could manage excessive fecal and serum TG levels.

### 3.6. Effect of Lr263 Supplementation on Tissue Weight at the End of the Experiment

Hamsters were killed after 6 weeks of Lr263 supplementation; liver, kidney, heart and epididymal fat pad (EFP) were removed and tissue weight was recorded for evaluating body composition. Kidney and heart weight and relative kidney and liver weight (%) did not differ among groups (Table 2).

The liver and EFP weight significantly differed among groups (F(4,35) = 66.07, *p* < 0.05, η2 = 0.883; F(4,35) = 4.22, *p* < 0.05, η2 = 0.325, respectively) and was higher with HCD alone, by 1.71- and 1.40-fold (*p* < 0.0001), than controls. The liver weight was lower with Lr263-1X, Lr263-2X and Lr263-5X supplementation [8.4% (*p* = 0.0045), 8.4% (*p* = 0.0047) and 10.4% (*p* = 0.0006), respectively] than HCD alone. In a previous study, liver weight significantly increased with a high-cholesterol diet [31]. We found that Lr263 supplementation could decrease the increased liver weight caused by a high cholesterol diet. The EFP weight was lower with Lr263-1X, Lr263-2X and Lr263-5X (8.4% (*p* = 0.0045), 8.4% (*p* = 0.0047) and 10.4% (*p* = 0.0006), respectively) than HCD alone. EFP weight was less with Lr263-2X and Lr263-5X (19.7% (*p* = 0.0104) and 19.2% (*p* = 0.012), respectively) than HCD alone. A similar study, involving kimchi lactic acid bacteria, showed significantly reduced TG and TC levels in liver and epididymal adipose tissue in a high-cholesterol diet model [32]. We also showed that Lr263 had consistent physiological activities on TG and TC content and fat composition under a hyperlipidemic model.

**Table 2:** Tissue weights at the end of the experiment.

Organ Weight	Control	HCD	Lr263-1X	Lr263-2X	Lr263-5X	Trend Analysis
Liver (g)	3.50 ± 0.37 ^a^	5.97 ± 0.28 ^c^	5.47 ± 0.32 ^b^	5.47 ± 0.37 ^b^	5.35 ± 0.31 ^b^	0.0003
Kidney (g)	1.17 ± 0.05	1.14 ± 0.05	1.15 ± 0.06	1.17 ± 0.08	1.13 ± 0.07	0.8553
Heart (g)	0.53 ± 0.04	0.50 ± 0.05	0.52 ± 0.06	0.53 ± 0.02	0.50 ± 0.03	0.8219
EFP (g)	2.12 ± 0.34 ^a^	2.98 ± 0.59 ^b^	2.56 ± 0.27 ^ab^	2.40 ± 0.49 ^a^	2.41 ± 0.40 ^a^	0.0095
Relative liver (%)	2.84 ± 0.19 ^a^	4.76 ± 0.52 ^c^	4.25 ± 0.39 ^b^	4.43 ± 0.25 ^bc^	4.48 ± 0.43 ^bc^	0.6720
Relative kidney (%)	0.96 ± 0.08	0.91 ± 0.07	0.89 ± 0.03	0.95 ± 0.07	0.95 ± 0.11	0.3636
Relative heart (%)	0.43 ± 0.04	0.40 ± 0.05	0.40 ± 0.04	0.43 ± 0.02	0.42 ± 0.03	0.0321
Relative EFP (%)	1.71 ± 0.16 ^a^	2.38 ± 0.56 ^b^	1.98 ± 0.19 ^a^	1.92 ± 0.25 ^a^	2.01 ± 0.28 ^a^	0.1194

Data are mean ± SD, *n* = 8 hamsters per group. Values in the same row with different superscripts letters (a, b, c) significantly differ at *p* < 0.05 by one-way ANOVA. EFP: Epididymal fat pad.

### 3.7. Effect of Lr263 Supplementation on Biochemical Analyses at the End of the Experiment

**Figure 7:**
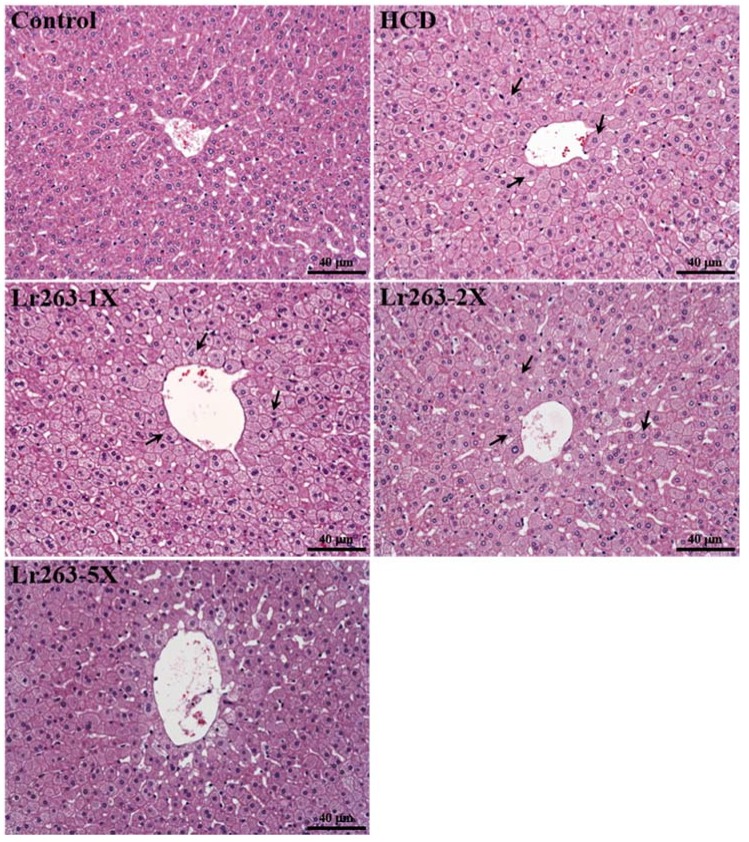
Effect of six-week supplementation with Lr263 on morphology of liver tissues in hyperlipidemic hamsters. Arrows indicate fat droplets. Specimens were photographed by light microscopy. (H & E stain, magnification: ×200, Scale bar, 40 μm)

In the present study, we observed the beneficial effects of Lr263 on indicators of lipid-lowering capacity. We further investigated whether six-week Lr263 treatment had any negative effect on other biochemical markers of hamsters. We examined the tissue- and health status-related biochemical parameters and major organs including liver, heart, kidney, and lung by histopathology (Table 3 and Figure 7). Supplementation of Lr263 for six weeks had no adverse effects. The ALT index significantly differed among groups (F(4,35) = 7.168, *p* < 0.05, η2 = 0.450) and the HCD diet increased the ALT index (*p* < 0.0001) as compared with controls. For clinical application, statins, which are cholesterol-lowering drugs, affect all aspects of the cholesterol profile, but all have been shown to significantly elevate liver enzyme levels [33]. We found that Lr263 supplementation significantly decreased the ALT index and had dose-dependent effects on trend analysis (*p* = 0.0143). Therefore, Lr263 supplementation could provide alternative nutrient supplementation to ameliorate the side effects of statins and has a potential effect on lowering hyperlipidemia.

### 3.8. Effect of Lr263 Supplementation on Histology at the End of the Experiment

Liver tissue from hamsters fed a normal chow diet showed a clear hepatic cord and sinusoid (Figure 7). In a previous study, the high-fat diet-induced pathological morphology in livers significantly differed in rodent species. The fat was microvesicular in hamsters and mixed (macro- and microvesicular) in mice [34]. In the HCD-fed group, fatty liver changes (steatosis) were detected in all animals, with hepatocytes comprising microvesicles filled with small lipid droplets, which is similar with the previous pathological observation. The degree of fatty change was significantly lower in the Lr-263-5X than HCD-fed group, with no significant difference in steatosis status between Lr-263-1X, Lr-263-2X and HCD-fed groups.

**Table 3:** Biochemical analysis of the *L.* *reuteri* 263 (Lr263) treatment groups at the end of experiment.

Parameters	Control	HCD	Lr263-1X	Lr263-2X	Lr263-5X	Trend Analysis
AST (U/L)	48 ± 10	46 ± 11	45 ± 8	43 ± 4	44 ± 4	0.8777
ALT (U/L)	72 ± 9 ^a^	101 ± 15 ^c^	90 ± 11 ^bc^	89 ± 12 ^b^	87 ± 5 ^b^	0.0143
LDH (U/L)	139 ± 25	142 ± 16	137 ± 16	138 ± 16	134 ± 13	0.3844
Albumin (g/dL)	3.6 ± 0.2	3.5 ± 0.2	3.6 ± 0.2	3.6 ± 0.2	3.6 ± 0.1	0.8501
TP (g/dL)	5.6 ± 0.3	5.5 ± 0.2	5.8 ± 0.2	5.7 ± 0.2	5.6 ± 0.2	0.8501
BUN (mg/dL)	17.9 ± 2.3	17.8 ± 1.8	17.9 ± 3.2	17.6 ± 0.8	17.7 ± 1.8	0.7215
Creatinine (mg/dL)	0.12 ± 0.03	0.12 ± 0.02	0.13 ± 0.06	0.12 ± 0.04	0.12 ± 0.02	0.6189
Glucose (mg/dL)	99 ± 12 ^a^	151 ± 25 ^b^	110 ± 17 ^a^	103 ± 21 ^a^	98 ± 14 ^a^	<0.0001

Data are mean ± SD for *n* = 8 hamsters per group. Values in the same row with different superscripts letters (a, b, c) significantly differ at *p* < 0.05 by one-way ANOVA. AST, aspartate aminotransferase; ALT, alanine aminotransferase; LDH, lactate dehydrogenase; TP, total protein; BUN, blood urea nitrogen.

## 4. Conclusions

Lr263 has lipid-lowering actions by decreasing serum TG and TC levels, liver TG and TC levels, fecal TG levels and serum LDL-C and LDL-C/HDL-C levels in hyperlipidemic hamsters. We found that six-week Lr263 supplementation significantly improved the hyperlipidemia syndrome in hamsters. Lr263 increased HDL-C levels to decrease the LDL-C/HDL-C ratio, which is beneficial to human health by reducing the risk for developing cardiovascular disease. In biochemical study, we found no gross abnormalities attributed to Lr263 treatment. Many studies demonstrate the *L. reuteri* has antioxidant activity and immune functions [35,36]. The possible mechanism for reducing serum cholesterol by *L. reuteri* is activating bile salt hydrolase enzyme to increase bile acid excretion [37,38]. In clinical studies, *L. reuteri* significantly reduced LDL-C level and is considered an option to prevent cardiovascular disease [39]. In conclusion, our study provides experiment-based evidence to support that Lr263 may have potential as a therapeutic for reducing blood lipid levels and lowering hyperlipidemic effects.
